# Deciphering the BSE-type specific cell and tissue tropisms of atypical (H and L) and classical BSE

**DOI:** 10.1080/19336896.2019.1651180

**Published:** 2019-09-03

**Authors:** Anne Balkema-Buschmann, Grit Priemer, Reiner Ulrich, Romano Strobelt, Bob Hills, Martin H. Groschup

**Affiliations:** a Friedrich-Loeffler-Institut, Institute of Novel and Emerging Infectious Diseases, Greifswald, Germany; b Department of Experimental Animal Facilities and Biorisk Management, Friedrich-Loeffler-Institut, Greifswald, Germany; c Health Canada, Transmissible Spongiform Encephalopathy Secretariat, Ottawa, Ontario, Canada

**Keywords:** Atypical BSE, central nervous system, peripheral nervous system, neurons, glial cells, origin of BSE, PrP^Sc^, cattle

## Abstract

After the discovery of two atypical bovine spongiform encephalopathy (BSE) forms in France and Italy designated H- and L-BSE, the question arose whether these new forms differed from classical BSE (C-BSE) in their pathogenesis. Samples collected from cattle in the clinical stage of BSE during an intracranial challenge study with L- and H-BSE were analysed using biochemical and histological methods as well as in a transgenic mouse bioassay. Our results generally confirmed what had been described for C-BSE to be true also for both atypical BSE forms, namely the restriction of the pathological prion protein (PrP^Sc^) and BSE infectivity to the nervous system. However, analysis of samples collected under identical conditions from both atypical H- and L-BSE forms allowed us a more precise assessment of the grade of involvement of different tissues during the clinical end stage of disease as compared to C-BSE. One important feature is the involvement of the peripheral nervous and musculoskeletal tissues in both L-BSE and H-BSE affected cattle. We were, however, able to show that in H-BSE cases, the PrP^Sc^ depositions in the central and peripheral nervous system are dominated by a glial pattern, whereas a neuronal deposition pattern dominates in L-BSE cases, indicating differences in the cellular and topical tropism of both atypical BSE forms. As a consequence of this cell tropism, H-BSE seems to spread more rapidly from the CNS into the periphery via the glial cell system such as Schwann cells, as opposed to L-BSE which is mostly propagated via neuronal cells.

## Introduction

Bovine spongiform encephalopathy (BSE) in cattle belongs to the group of transmissible spongiform encephalopathies (TSEs), also called ‘prion diseases’. These inevitably fatal neurodegenerative disorders can affect numerous mammalian species. Hallmarks of TSEs are spongiform changes in the central nervous system (CNS) [1] and the accumulation of a pathological prion protein (PrP^Sc^), predominantly in the nervous system. Classical BSE (C-BSE) in cattle has been shown to be causatively linked to the feeding of BSE-contaminated meat and bone meal and milk replacers [2,3]. The prohibition of these feeding practices has effectively stopped the circulation of the BSE agent in the feed chain of bovines and drastically reduced the infection rate of cattle born after the implementation of these measures.

For C-BSE, the general restriction of the PrP^Sc^ accumulation and infectivity to the central nervous system of clinically affected cattle is well acknowledged [4–6]. The brainstem at the level of the obex with the nuclei of the solitary tract, the spinal tract of the trigeminal nerve as well as the dorsal motor nucleus of the vagus nerve (DMNV) are already involved during the late preclinical stage of the disease [7–9]. However, in the majority of cases, the spinal cord only displays vacuolar degeneration and mild PrP^Sc^ depositions after the animal has entered the clinical stage of the disease. In addition, BSE infectivity has been detected in the peripheral nervous system (PNS) and in the adrenal gland of cattle naturally infected or experimentally challenged with classical BSE by using a highly sensitive transgenic mouse bioassay [10]. Minor amounts of infectivity have also been demonstrated in a *Musc. semitendinosus* sample of a natural C-BSE case in the final disease stage by transgenic mouse bioassay [6]. More recently, the analysis of peripheral, including non-nerval, tissues from cattle orally challenged with C-BSE by protein misfolding cyclic amplification (PMCA) also revealed the presence of PrP^Sc^ in tissues of the digestive tract [11]. Studies on the C-BSE pathogenesis after oral challenge revealed that the uptake of infectivity in the Peyer’s patches of the small intestine is followed by transfer to the enteric nervous system and transport along the mesenteric ganglion/coeliac ganglion complex and the splanchnic nerves to the sympathetic or parasympathetic nerve bundles and/or the spinal cord, and finally to the brainstem [11–17].

The lymphoreticular system (LRS) of BSE-affected cattle was found to be generally free of detectable PrP^Sc^ and infectivity, with only the tonsils [18], the Peyer´s patches of the gut [19] and the mesenteric lymph node [11] representing exceptions that most likely play a role in the uptake of the agent after an oral infection. In contrast, PrP^Sc^ and infectivity are easily detected throughout the peripheral nervous system and the LRS of scrapie-affected sheep and goats [20,21].

The aetiology as well as the pathogenesis of both atypical BSE forms, designated H- and L-BSE are less understood so far, due to their relatively late discovery in France and Italy [22,23], and due to the fact that only very few samples of natural H- or L-BSE cases have been available for analysis. Efforts were made to investigate the agent distribution in clinically affected cattle experimentally challenged with atypical BSE by intracranial inoculation. Oral challenge of cattle with atypical BSE has proved to be highly inefficient [24 and S. Czub, personal communication].

We recently challenged cattle intracranially with H- and L-BSE [25]. First results of this study already indicated that PrP^Sc^ was detectable in the central nervous system (CNS) by biochemical methods (BSE rapid test as well as precipitation with phosphotungstic acid (PTA) followed by western blot), whereas samples from the peripheral tissues, including the PNS and LRS, remained negative [25]. In other studies, atrophic alterations of the muscular tissue [26] as well as PrP^Sc^ deposits and infectivity in the skeletal muscle of H-BSE and L-BSE affected cattle were observed [27]. In another L-BSE transmission study, Western blot analysis revealed a PrP^Sc^ accumulation in peripheral nervous samples, while no PrP^Sc^ was detectable in any lymphoid tissues [28]. Other authors reported PrP^Sc^ accumulations in peripheral tissue samples of intracranially challenged cattle, precisely in muscle spindles and in the trigeminal ganglion, as detected by immunohistochemical analysis, while the LRS and the enteric nervous system (ENS) displayed no involvement [29]. Taken together, the LRS is definitely not involved in the pathogenesis and agent distribution in either of the atypical BSE forms, while the peripheral nervous system as well as skeletal muscles have been shown to contain PrP^Sc^ and infectivity in the late stages of both atypical BSE forms [27,29,30].

In this present study, we further analysed peripheral samples collected from cattle that were at different stages of the clinical phase of disease after intracranial challenge with H- or L-BSE [25] and compared these findings to those in samples collected from cattle after oral challenge with C-BSE [13]. This is also relevant regarding the current definition of specified risk materials (SRMs), which is based on the classical BSE pathogenesis. Although different routes of inoculation were used in both studies, results from earlier studies comparing both inoculation routes indicate – with all necessary reservations – a very similar pathogenesis after oral and intracranial challenge [31]. To the best of our knowledge, this is the first attempt for a direct comparison of all three BSE forms regarding their PrP^Sc^ and BSE infectivity distribution in cattle.

## Results

### Biochemical analysis

All tissue samples of animal RA01 sacrificed 5 month after challenge with L-BSE and which was in the preclinical stage of the disease, were negative in all biochemical tests that were performed. From the cattle challenged with H-BSE, no animal was sacrificed in the preclinical phase.

#### IDEXX HerdChek® BSE EIA

For all animals sacrificed at the clinical stage of the disease (12 mpi to 16 mpi), the obex and the spinal cord samples gave strong positive results in the IDEXX BSE EIA (OD values >2.0). The optic nerve samples were also detected positive for all animals sacrificed between 14 and 16 mpi, with slightly higher OD values (1.266 on average) for the samples collected from H-BSE infected cattle as compared to 0.896 on average for the L-BSE infected animals (Table 1). Peripheral tissue samples including the PNS and LRS gave OD values below the cutoff value of about 0.089 on average (SD = 0.01) for both atypical BSE types.

**Table 1.** Neuronal tissues analysed by biochemical and histological methods.

Time post inoculation	12 mpi			14 mpi			15 mpi			16 mpi			16 mpi		
**L-type BSE/** **Animal ID**	**RA 05**			**RA 06**			**RA 03**			**RA 02**			**RA 04**		
**Sampled tissue/** **Testing method**	**IDEXX**	**PTA-WB**	**IHC**	**IDEXX**	**PTA-WB**	**IHC**	**IDEXX**	**PTA-WB**	**IHC**	**IDEXX**	**PTA-WB**	**IHC**	**IDEXX**	**PTA-WB**	**IHC**
**obex/cranial Medulla**	3.071	pos 1:128	+	3.182	pos 1:128	+	3.191	pos 1:256	++	3.103	pos 1:1024	+++	3.195	pos 1:512	+++
**spinal cord T7**	1.591	-	+	2.363	pos 1:16	+	3.251	pos 1:32	++	3.097	pos 1:8	++	3.218	pos 1:16	++
**spinal cord L3**	3.0	pos 1:8	+	3.21	pos 1:16	+	3.23	pos 1:32	++	3.278	pos 1:1	++	3.261	pos 1:32	++
**dorsal root ganglia**	-	-	-	-	-	(+)	-	-	(+)	-	-	+	-	-	-
**trigeminal ganglia**	-	-	-	-	-	(+)	-	-	-	-	-	-	-	-	-
**optic nerve**	-	-	(+)	0.892	pos 1:8	(+)	0.628	pos 1:1	+	0.426	pos 1:1	+	2.377	pos 1:64	+
**cervical vagus nerve**	-	-	-	-	-	-	-	-	+	-	-	-	-	-	(+)
**stellate ganglia**	-	-	-	-	-	-	-	-	+	-	-	-	-	-	-
**H-Type BSE/** **Animal ID**	**RA 10**			**RA 14**			**RA 13**			**RA 15**			**RA 16**		
obex/cranial Medulla	3.061	pos 1:32	++	3.136	pos 1:64	++	3.147	pos 1:16	++	3.09	pos 1:32	++	3.114	pos 1:32	+
**spinal cord T7**	1.39	pos 1:1	+	2.140	pos 1:4	++	1.429	pos 1:1	++	2.704	pos 1:2	++	2.28	pos 1:4	+
**spinal cord L3**	2.217	pos 1:32	+	2,770	pos 1:4	++	2.95	-	++	1.237	pos 1:32	+	2.7	pos 1:1	++
**dorsal root ganglia**	-	-	(+)	-	-	-	-	-	*+*	-	-	+	-	-	+
**trigeminal ganglia**	-	-	(+)	-	-	(+)	-	-	(+)	-	-	-	-	-	+
**optic nerve**	-	-	+	1.818	pos 1:4	+	0.704	pos 1:1	+	2.224	pos 1:8	++	1.396	-	n.d.
**cervical vagus nerve**	-	-	(+)	-	-	-	-	-	+	-	-	+	-	-	+
**stellate ganglia**	-	-	-	-	-	-	-	-	+	-	-	+	-	-	+

- negative; (+) weak positive; + positive, ++ moderate positive; +++ strong positive

#### PTA-WB

PTA-WB of the CNS samples was positive in dilutions up to 1:1024 for all 10 animals challenged with H- and L-BSE that had been in the clinical stage of the disease (Table 1). The optic nerve samples were negative for the two animals that were sacrificed at 12 mpi, but were tested positive (in dilution up to 1:64; Table 1) in animals sacrificed between 14 and 16 mpi. All other tissue samples of the peripheral nervous system (PNS), the LRS, as well as the musculoskeletal, alimentary, respiratory, or the reproductive system were negative in L-BSE and H-BSE affected cattle using the routine biochemical analysis.

**Figure 1.**
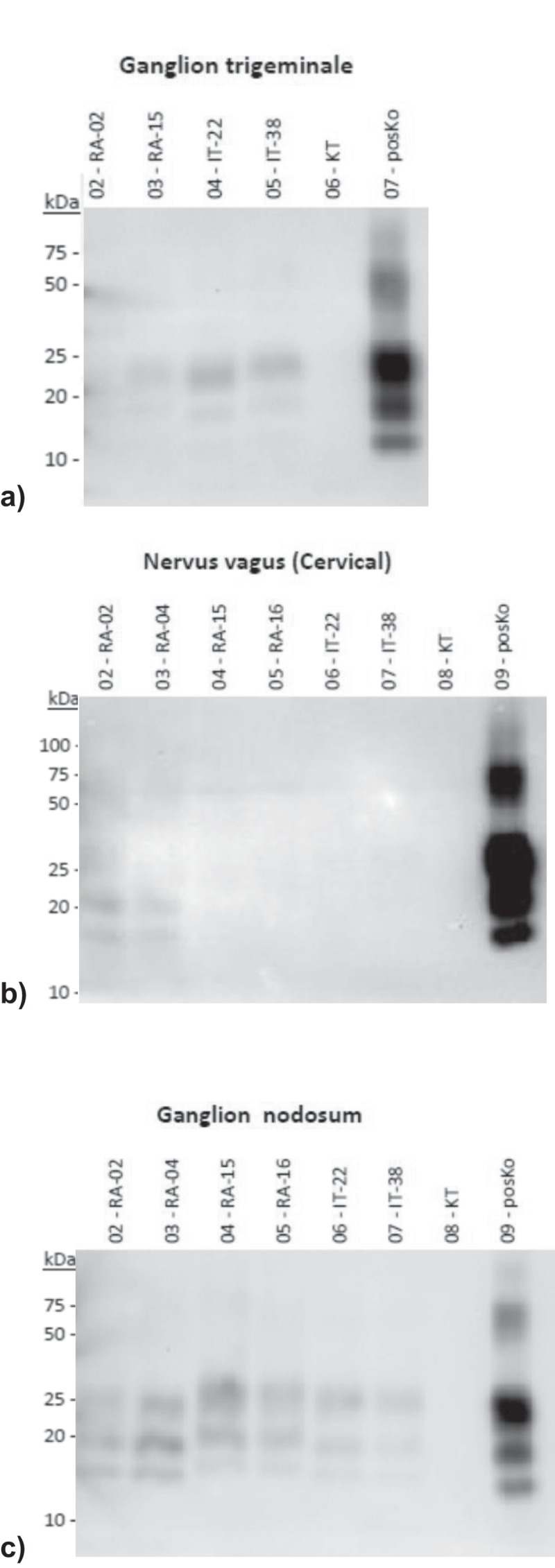
Detection of PrP^Sc^ deposition in selected tissues of the central and peripheral nervous system as revealed by ultracentrifugation assay (UC-A) and Western blot illustrating (a) low amount of PrP^Sc^ in the trigeminal ganglion samples from H-BSE and C-BSE affected cattle, while the same samples collected from L-BSE affected cattle were negative, (b) cervical vagus nerve samples giving weak positive results for cattle affected with L-BSE and while being negative in the case of H-BSE and C-BSE affected cattle, (c) nodose ganglion samples of cattle affected with all three BSE forms were positive in the UC-A analysis.RA = cattle experimentally challenged with atypical BSEIT = cattle experimentally challenged with classical BSEKT = mock infected negative control cattle.

To further address the role of the different parts of the nervous system in the propagation and transport of BSE prions, we analysed a set of samples (Supplementary Table 1) collected from two animals of each inoculation group (H-, L- and C-BSE) with a highly sensitive PrP^Sc^ purification protocol using ultracentrifugation followed by Western blot (hence referred to as ultracentrifugation assay; UC-A). Doing so, we revealed low amounts of PrP^Sc^ in the trigeminal ganglion samples from H-BSE affected cattle, while the same samples collected from L-BSE affected cattle were negative (Figure 1(a)). Meanwhile, the cervical vagus nerve samples gave weak positive results for cattle affected with L-BSE and were negative in the case of H-BSE affected cattle (Figure 1(b)). Stellate ganglion samples of both L-BSE and H-BSE challenged cattle were negative in this assay, while PrP^Sc^ was detected in all analysed samples of the nodose ganglion (Figure 1(c)).

### Histopathological and immunohistochemical analysis

For immunohistochemical analysis, the mab cocktail 6C2/F99 was applied in order to benefit from a synergistic effect on the sensitivity of two PrP-specific antibodies. To verify the results obtained with the mab cocktail 6C2/F99, we repeated the IHC staining procedure on the samples listed in Supplementary Table 1 from two animals per BSE type (RA02, RA04, RA15, RA16) using mab 6C2 alone. This confirmed the results obtained using the mab cocktail.

**Figure 2.**
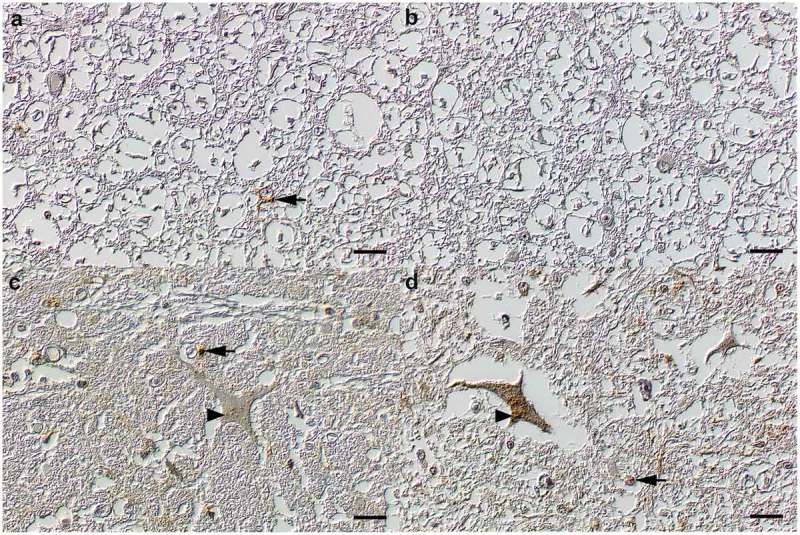
Spinal cord. (a) H-BSE (RA 16) spinal cord, white matter. Note the rare cytoplasmic granular PrP^Sc^ immunoreactivity in glial cells (arrow). (b) L-BSE (RA 02) spinal cord, white matter. The white matter shows no PrP^Sc^ immunoreactivity. (c) H-BSE (RA 16) spinal cord, grey matter. Minor multifocal granular PrP^Sc^ depositions within the neuropil (arrow), and rare oligofocal granules within neurons (arrowhead). (d) L-BSE (RA 02) spinal cord, grey matter. Moderate, multifocal, cytoplasmic, finely granular PrP^Sc^ immunoreactivity within neurons (arrowhead) and a faint multifocal reaction in the neuropil (arrow). Immunohistochemistry; monoclonal anti-PrP antibody cocktail (clones: 6C2 and F99/97.6.1); horseradish peroxidase-and polymer coupled secondary rabbit anti-mouse antibody (EnVision^TM^, DAKO); diaminobenzidine tetrahydrochloride (brown); Mayer`s hematoxylin counterstain (blue); Nomarski contrast; Bars = 20 µm.

All obex and spinal cord samples were tested positive from all animals that were sacrificed after 12–16 mpi. For H-BSE affected animals, the PrP^Sc^ accumulation pattern in the obex, especially in the nucleus of the solitary tract, the nucleus of the spinal tract of the trigeminal nerve and the dorsal motor nucleus of the vagus nerve (DMNV) as well as the spinal cord was dominated by a glial, coarse granular PrP^Sc^ deposition pattern. In contrast, the samples from L-BSE affected cattle majorly displayed intraneuronal depositions. Corresponding deposition patterns were determined for both the thoracic (T7) and the lumbal segments (L3) of the spinal cord. Interestingly in H-BSE affected cattle, only few individual glial cells within the white matter of the spinal cord displayed immunoreactivity (Figure 2(a)). In contrast, L-BSE affected cattle only showed faint or no PrP^Sc^ immunolabelling in the white matter of the spinal cord, but showed a moderate labelling in neurons of the grey matter (Figure 2(d)).

For H-BSE affected cattle, we detected PrP^Sc^ depositions in the dorsal root ganglia (DRG) of the animal sacrificed at 12, 15 and 16 mpi, while no PrP^Sc^ was detectable in the animal sacrificed at 14 mpi, which may well be due to individual variation. The DRG in L-BSE affected cattle were consistently detected positive from 14 to 16 mpi. Once again, in H-BSE affected cattle we observed a glial deposition pattern (5–10% of the analysed cells) with a low number of positive neurons (<1%), while for L-BSE, we only determined individual neurons or glial cells with PrP^Sc^ deposition (both below 5% of analysed cells).

**Figure 3.**
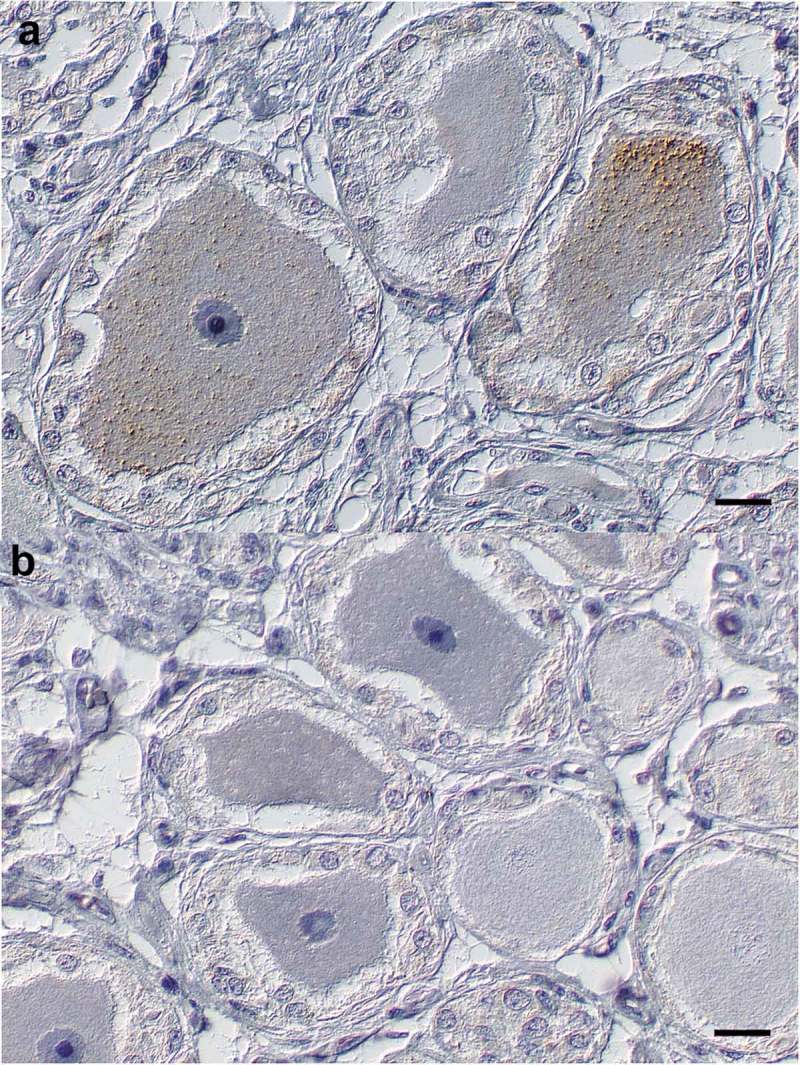
Trigeminal ganglion. (a) H-BSE (RA 16). Oligofocal ganglionic neurons and rare satellite cells display mild to moderate cytoplasmic accumulations of coarse PrP^Sc^ immunoreactive granula. (b) L-BSE (RA 03). Ganglionic neurons and satellite cells display no PrP^Sc^ immunoreactivity. Immunohistochemistry; monoclonal anti-PrP antibody cocktail (clones: 6C2 and F99/97.6.1); horseradish peroxidase-and polymer coupled secondary rabbit anti-mouse antibody (EnVision^TM^, DAKO); diaminobenzidine tetrahydrochloride (brown); Mayer`s hematoxylin counterstain (blue); Nomarski contrast; Bars = 20 µm.

**Figure 4.**
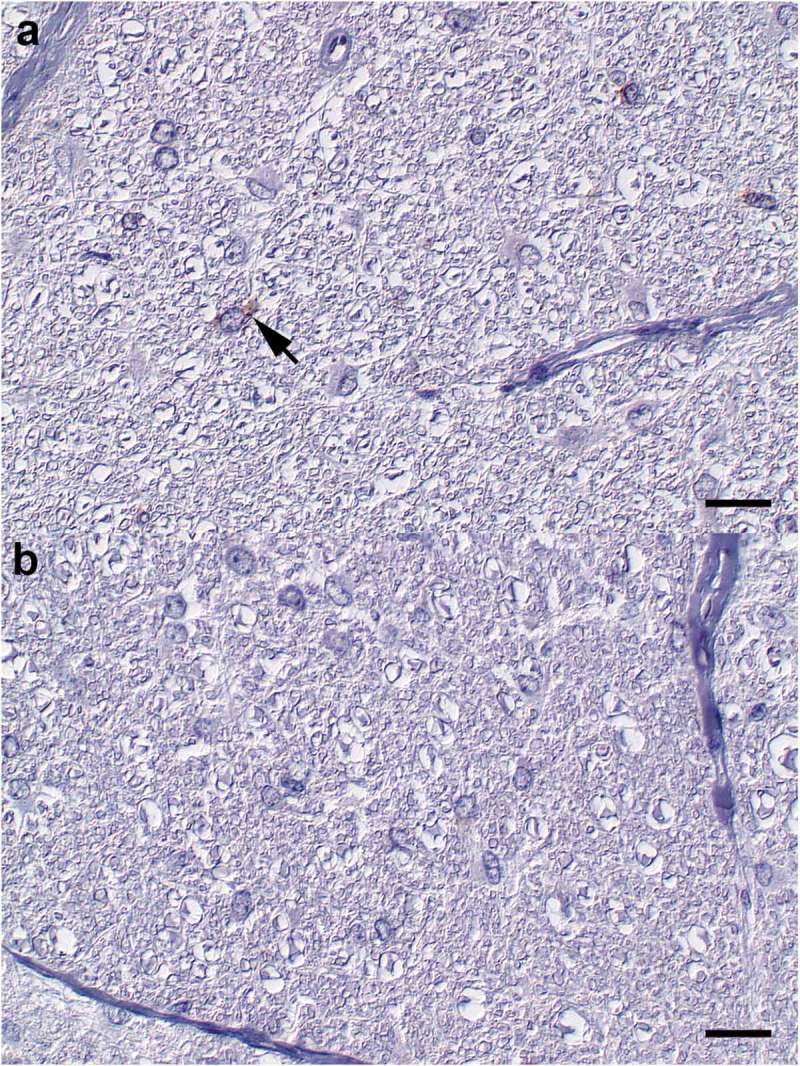
N. opticus. (a) H-BSE (RA 16). Oligofocal Schwann cells show cytoplasmic accumulations of few PrP^Sc^ immunoreactive granula (arrow). (b) L-BSE (RA 03). Axons and Schwann display no PrP^Sc^ immunoreactivity. Immunohistochemistry; monoclonal anti-PrP antibody cocktail (clones: 6C2 and F99/97.6.1); horseradish peroxidase-and polymer coupled secondary rabbit anti-mouse antibody (EnVision^TM^, DAKO); diaminobenzidine tetrahydrochloride (brown); Mayer`s hematoxylin counterstain (blue); Nomarski contrast; Bars = 20 µm.

While all H-BSE infected cattle exhibited mild PrP^Sc^ accumulation in the trigeminal ganglia, we could detect only traces of PrP^Sc^ accumulation in the trigeminal ganglion of one cattle challenged with L-BSE (Figure 3). The optic nerve was also tested positive by immunohistochemistry in cattle challenged with both atypical BSE forms from 12 mpi. Here, a glial PrP^Sc^ accumulation pattern was predominant, with bold glial pattern in H-BSE infected cattle and only faint deposits in L-BSE challenged cattle (Figure 4(a,b)). Analysis of the cervical part of the vagus nerve revealed weak positive results for the H-BSE affected animal sacrificed at 12 mpi, and for the animals sacrificed at 15 and 16 mpi for both H- and L-BSE. Finally, we detected PrP^Sc^ in the samples from the stellate ganglion for all three animals affected with H-BSE sacrificed at 15 and 16 mpi (RA13, RA15, RA16) and for one L-BSE affected animal sacrificed at 15 mpi (RA03) (Table 3).

**Table 2.** Bioassay results (attack rate and mean incubation time) in bovine PrP transgenic mice (Tgbov XV).

Tissue	L-BSE 16 mpi RA02	L-BSE 16 mpi RA04	H-BSE 16 mpi RA15	H-BSE 16 mpi RA16	C-BSE oral infection clinical end stage
Obex	14/14 177 d	12/12 182 d	8/12 304 d	14/15 299 d	14/14 208 d [17]
Dorsal root ganglion	15/15 332 d	6/15 378 d	4/15 632 d	7/14 460 d	n.d.
Cervical cranial ganglion	13/14 337 d	n.d.	1/15 504 d	1/14 530 d	12/13 281 d [33]
Trigeminal ganglion	13/15 287 d	15/15 286 d	8/14 441 d	8/15 443 d	14/14 305 d [44]
Stellate ganglion	11/12 266 d	15/15 250 d	13/14 467 d	9/14 487 d	8/8 337 d [17]
N. vagus pars cervicalis	9/15 344 d	14/15 343 d	7/15 533 d	7/13 401 d	8/8 339 d * [33]
N. saphenus	9/15 346 d	12/15 337 d	5/15 573 d	1/15 509 d	n.d.
N. medianus	10/15 365 d	14/15 336 d	0/12 > 731	9/15 529 d	n.d.
Musc. semitend.	5/13 378 d	4/11 399 d	0/15 > 735 d	3/11 455 d	1/10 520 d [17]
Musc. ligualis	0/10 > 540 d	0/9 > 729 d	0/9 > 735 d	0/14 > 735 d	13/14 390 d [44]
Palatine tonsils	0/14 > 749 d	0/14 > 742 d	0/14 > 732 d	0/9 > 728 d	0/11 > 706 d [17]
Ileal Peyers Patches	0/14 > 749 d	0/10 > 742 d	0/15 > 707 d	0/15 > 728 d	3/13 574 d [17]
Ileocaecal junction	0/13 > 735 d	0/11 > 735 d	0/12 > 735 d	0/15 > 728 d	n.d.
Ln. retropharyng.	0/13 > 740 d	0/15 > 742 d	0/14 > 732 d	0/14 > 728 d	n.d.
Spleen	0/14 > 728 d	0/15 > 728 d	0/15 > 728 d	0/12 > 728 d	0/14 > 727 d [17]

n.d. = not done

**Table 3.** Summary of PrP^Sc^ and infectivity detection in CNS and peripheral tissue samples.

	H-type BSE intracranial challenge, clinical end stage		L-type BSE intracranial challenge, clinical end stage		C-type BSE oral challenge, clinical end stage	
Tissue sample	**Infectivity**	**PrP^Sc^**	**Infectivity**	**PrP^Sc^**	**Infectivity**	**PrP^Sc^**
**Central nervous tissue (CNS)**						
**Obex**	+	+	+	+	+ [6; 17]	+
**Spinal cord (T7, L3)**	n.d.	+	n.d.	+	+ [6;17]	+
**Dorsal root ganglion (DRG)**	+	(+)	+	+	n.d.	+ [11; 44]
**Trigeminal ganglion**	+	+	+	(+)	+ [17]	+ [17]
**Peripheral nervous tissue (PNS)**						
**Stellate ganglion**	+	+	+	+	+ [17]	+ [11;44]
**Coeliac ganglion**	n.d.	-	n.d.	-	+ [17]	+ [17]
**Cervical vagus nerve**	+	+	+	+	+ [17]	+ [17]
**Optic nerve**	n.d.	+	n.d.	+	+ [6]	+ [11; 44]
**Facial nerve**	n.d.	-	n.d.	-	+ [6]	- [11; 44]
**Medial nerve**	n.d.	-	n.d.	-	- [6]	- [11; 44]
**Radial nerve**	n.d.	-	n.d.	-	- [17]	- [11;44]
**Saphenous nerve**	+	-	+	-	n.d.	- [11; 44]
**Tibial nerve**	n.d.	-	n.d.	-	n.d.	- [11; 44]
**Lymphoreticular System (LRS)**						
**Spleen**	-	-	-	-	- [6]	-
**Retropharyngeal lymph node**	-	-	-	-	n.d.	n.d.
**Mediastinal lymph node**	n.d.	-	n.d.	-	n.d.	- [11; 44]
**Mesenteric lymph node**	n.d.	-	n.d.	-	- [17]	+ [11; 44]
**Mammary lymph node**	n.d.	-	n.d.	-	n.d.	n.d.
**Ileal Peyers patches**	-	-	-	-	+ [6]	+ [11; 44]
**Alimentary, Respiratory & Reproductive Systems**						
**Salivary gland**	n.d.	-	n.d.	-	n.d.	- [11; 44]
**Esophagus**	n.d.	-	n.d.	-	n.d.	+ [11; 44]
**Rumen**	n.d.	-	n.d.	-	n.d.	+ [11; 44]
**Abomasum**	n.d.	-	n.d.	-	n.d.	+ [11; 44]
**Caecum (GALT)**	n.d.	-	n.d.	-	n.d.	+ [11; 44]
**Colon (GALT)**	-	-	-	-	n.d.	+ [11; 44]
**Rectum (GALT)**	n.d.	-	n.d.	-	n.d.	+ [11; 44]
**Pancreas**	n.d.	-	n.d.	-	n.d.	- [11; 44]
**Adrenal gland**	n.d.	-	n.d.	-	n.d.	+ [11; 44]
**Mammary gland**	n.d.	-	n.d.	-	n.d.	- [11; 44]
**Uterus**	n.d.	-	n.d.	-	n.d.	- [11; 44]
**Ovary**	n.d.	-	n.d.	-	n.d.	- [11; 44]
**Gall bladder**	n.d.	-	n.d.	-	n.d.	- [11; 44]
**Lung**	n.d.	-	n.d.	-	n.d.	- [11; 44]
**Musculoskeletal System**						
**M. lingualis**	-	-	-	-	+ [16]	-
**Heart**	n.d.	-	n.d.	-	- [6]	- [11; 44]
**M. biceps brachii**	n.d.	-	n.d.	-	n.d.	- [11; 44]
**M. semitendinosus**	+	-	+	-	+ [6]	- [11; 44]
**M. psoas major**	n.d.	-	n.d.	-	n.d.	- [11; 44]
**M. longissimus dorsi**	n.d.	-	n.d.	(+)	- [6]	-

- negative; (+) weak positive; + positive; n.d. = not done

Negative control sections from the respective tissues of one of the mock-infected animals are presented in Supplementary Figure 1.

All other peripheral tissue samples of the lymphoreticular system, the alimentary, respiratory and reproductive system as well as from the musculoskeletal systems tested negative in this analysis.

**Figure 5.**
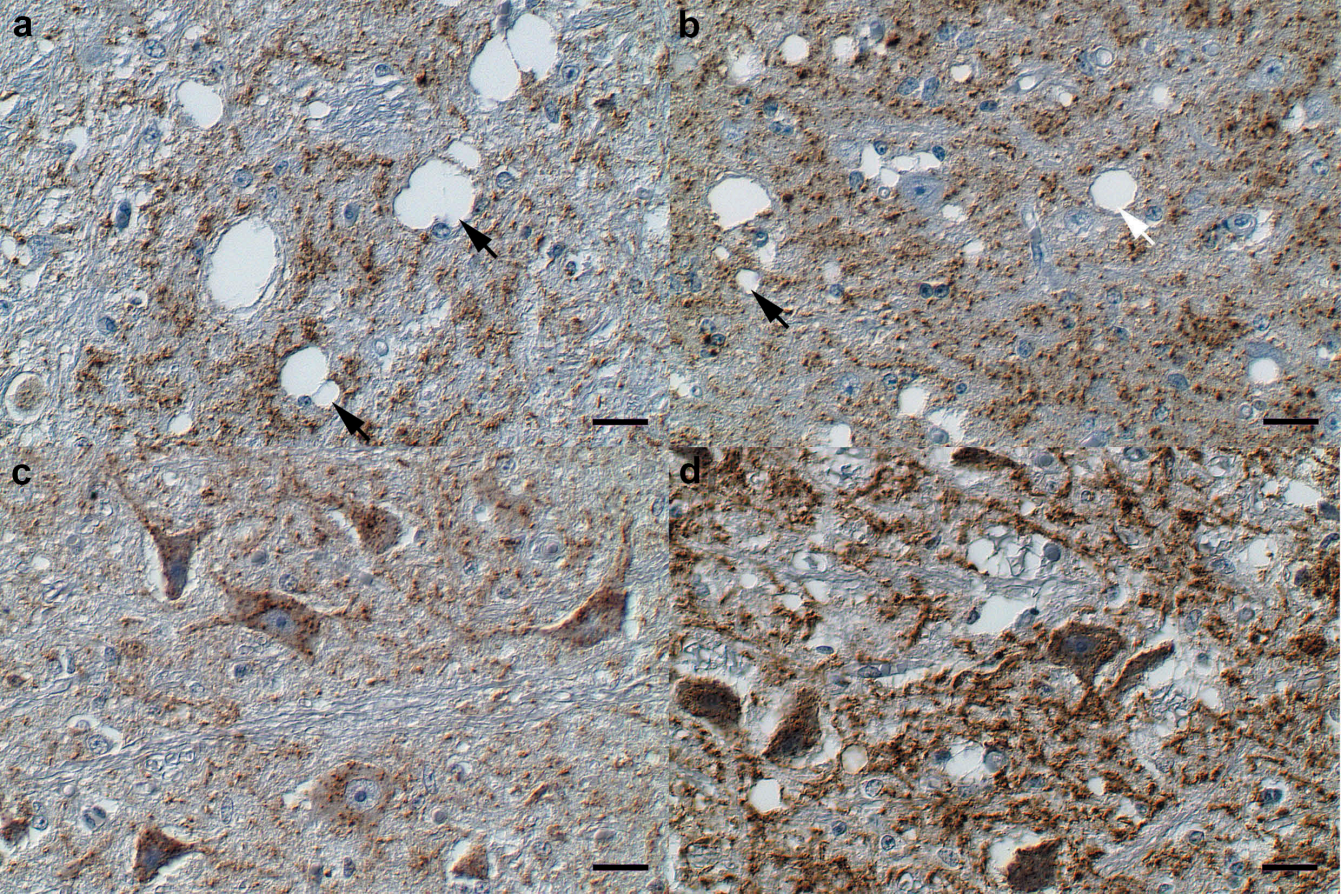
Marked BSE-type as well as locus specific variance in the amount of PrP^Sc^ depositions and vacuolations. (a) H-BSE (RA 16) brain, obex, nucleus of the spinal tract of the trigeminal nerve. Abundant glial cells and some neurons exhibit cytoplasmic, coarse granular PrP^Sc^ immunoreactive depositions. Multiple neurons exhibit large cytoplasmic vacuolations (arrows). (b) L-BSE (RA 03) brain, obex, nucleus of the spinal tract of the trigeminal nerve. Abundant glial cells and neurons exhibit cytoplasmic, coarse granular PrP^Sc^ immunoreactive depositions. The vacuoles are mostly intraparenchymal (arrow). (c) H-BSE (RA 13) brain, nucleus olivarius. Abundant, mostly intraneuronal, cytoplasmic, coarse granular PrP^Sc^ immunoreactive depositions but no vacuolations. (d) L-BSE (RA 02) brain, obex, nucleus olivarius. Abundant, mostly intraneuronal, cytoplasmic, coarse granular PrP^Sc^ immunoreactive depositions. Immunohistochemistry; monoclonal anti-PrP antibody cocktail (clones: 6C2 and F99/97.6.1); horseradish peroxidase-and polymer coupled secondary rabbit anti-mouse antibody (EnVision^TM^, DAKO); diaminobenzidine tetrahydrochloride (brown); Mayer`s hematoxylin counterstain (blue); Nomarski contrast; Bars = 20 µm.

By histopathological analysis, we were able to determine specific differences in the quality and quantity of vacuolar changes and PrP^Sc^ depositions between both atypical BSE forms during the course of the disease, especially at the late stage of disease (16 mpi). Cattle challenged with H-BSE showed generally low quantities of small vacuoles in the neuropil of the obex (Figure 5(a,c)), while L-BSE affected cattle mostly displayed numerous and large, partly confluent spongiform degenerations in the neuropil of the obex, especially in the solitary and trigeminal tract nuclei, and to a lesser extent in the dorsal motor nucleus of the vagus nerve, DMNV (Figure 5(b,d)). For L-BSE, the neurons of analysed ganglia (dorsal root ganglia, trigeminal ganglia) sometimes even displayed multiple vacuoles. Intraneuronal vacuolization in the obex region was negligible for both atypical BSE forms.

### Mouse bioassay in bovine PrP transgenic mice (Tgbov XV)

#### Parallel titration of H- and L-BSE in Tgbov XV mice

As published from an earlier transmission experiment of classical and atypical BSE into bovine PrP transgenic mice, challenge of Tgbov XV mice with obex samples collected from German H-BSE and L-BSE field cases had resulted in distinctly prolonged incubation times for H-BSE (330 days) and shorter incubation times for L-BSE (185 days) in comparison to 230 days that had been repeatedly determined for C-BSE in the same mouse model [32]. Similar incubation times were again observed in Tgbov XV mice after the intra-species passage of both atypical BSE field cases in cattle, namely 304 days (RA15) and 299 days (RA16) for H-BSE with attack rates of 67% and 93%, respectively, and 177 (RA02) and 182 days (RA04), both with 100% attack rates for L-BSE. Parallel titration experiment of H- and L-BSE in Tgbox XV revealed a titre of 10^−3,71^ LD_50_ for H-BSE and a titre of 10^−4,77^ LD_50_ for L-BSE, with generally longer incubation times in H-BSE infected mice (Supplementary Table 2). For comparison, the titre determined for a C-BSE-inoculum was 10^−5,73^ [33].

Incubation times in transgenic mice after inoculation of the trigeminal and stellate ganglia were prolonged by approximately 70% of the incubation time, and attack rates were slightly lower than after challenge with the obex samples from the same animals, indicative of a factor 10^3^ lower infectivity titre (as deduced from the titration experiments). Challenge of transgenic mice with samples from the dorsal root ganglion, the cervical cranial ganglion, the thoracic part of the vagus nerve, the *N. saphenus* and *N. medianus* from L BSE affected cattle resulted in incubation times and transmission rates that were prolonged by more than 80% and lower attack rates as compared to the obex sample, indicating infectivity titres that are by a factor 10^4^ or more lower than determined for CNS samples (Table 2, Supplementary Table 2).

Challenge of Tgbov XV mice with the same samples collected from H-BSE affected cattle resulted also in an incubation time increase by >80%, but with distinctly lower attack rates of below 50%, indicating a titre of at least 10^5^ lower than that determined for CNS samples (Table 3, Supplementary Table 2). We also detected very low amounts of infectivity in the *Musc. semitendinosus* samples from experimental cattle except RA15, with lower attack rates and longer incubation times than described for the other positive samples. Interestingly, Tgbov XV mice did not succumb to the disease after challenge with *Musc. lingualis* samples of cattle affected with atypical BSE after intracranial challenge, while we had shown in earlier studies that *Musc. lingualis* samples collected from cattle clinically affected with C-BSE upon oral challenge generally contained BSE infectivity [34].

In accordance with the results obtained for C-BSE in earlier studies, no infectivity was detectable in the samples of the lymphoreticular system that were analysed by transgenic mouse bioassay, which were the spleen, the palatine tonsils, the ileal and ileocaecal Peyer’s patches, as well as the retropharyngeal lymph nodes (Table 3).

## Discussion

Our biochemical, immunohistological and mouse bioassay results confirm the general restriction of PrP^Sc^ and infectivity to the nervous system, as already described for C-BSE [6] and for both atypical BSE forms in earlier, but less detailed studies that did not enable a direct comparison of both atypical BSE forms after experimental challenge [27–29,35,36]. However, we detected differences in the peripheral PrP^Sc^ and infectivity distribution patterns in the PNS for both atypical BSE forms, as assessed by immunohistochemistry, ultracentrifugation assay and transgenic mouse bioassay.

In this study, we decided to use mab cocktails in order to profit from additive effects of both applied antibodies. No negative effects concerning the use of a mab cocktail F89160.1.5/F99/97.6.1 as compared to the single mab F89/160.1.5 had been reported by others, and these authors suggested positive effects of an antibody cocktail in the recognition of more than one single epitope by one monoclonal antibody [37]. This was confirmed by our own findings for mab 6C2, which is highly sensitive either in a cocktail with mab F99/97.6.1, or when used as single mab. Furthermore, mab F99/97.6.1 has been reported to be very sensitive especially for the detection of H-BSE associated PrP^Sc^ [38]. These findings support observations reported by Okada et al. [35], who stated that intraglial PrP^Sc^ depositions seem to be hardly accessible for mabs binding to the core region of the prion protein.

As described earlier [25], the clinical presentation of the infected animals was dominated by depression and mild ataxia when left unmanipulated. However, upon targeted acoustic, tactile or optical stimuli, the animals developed a strong over-reactivity, as it is also seen in cattle affected with C-BSE. This behaviour may be caused by the fact that in both atypical BSE forms, the cortex areas of the brain are substantially more affected than in classical BSE [39], and the cortex is functionally involved in the regulation of the conscious awareness and the interpretation of sensation. Dysfunction of these areas may result in the observed depression. However, upon stimulus, the over-reactivity may be caused by a dysfunction of the brainstem, which is primarily involved in the control of the basic vital functions and the coordination of the 12 cranial nerves.

The biochemical analysis of representative tissue samples confirmed what had been described regarding the general restriction of PrP^Sc^ to the CNS and PNS samples for all three BSE forms. In our hands, skeletal muscle tissue samples were mostly negative for PrP^Sc^ deposition and infectivity in the case of both atypical BSE forms when analysed by IHC, ultracentrifugation assay and transgenic mouse bioassay. However, PrP^Sc^ was detected in　muscle　bundles　of　*Musc. longissimus* sample of an animal challenged with L-BSE (RA03), and we detected BSE infectivity in the *Musc. semitendinosus* sample from both L-BSE challenged cattle analysed in the mouse bioassay. This is confirmed by the results of other studies that revealed the involvement of skeletal muscles. Lombardi et al. [26] reported atrophy of skeletal muscle tissue and Suardi et al. [27] detected PrP^Sc^ accumulations in some skeletal muscles. The dissemination of PrP^Sc^ and infectivity via the neuronal pathway into skeletal muscle seems highly plausible in the case of L-BSE. Other studies revealed PrP^Sc^ depositions in muscle tissues of both H- and L-BSE affected cattle [29]. This could not be confirmed for H-BSE in our study, which might be due to the relatively early sacrifice of the challenged cattle after the onset of clinical signs.

Our analyses of samples collected from cattle challenged with all three BSE types and analysed using identical procedures allowed a more detailed comparative analysis of the deposition patterns. According to our IHC results, H-BSE infected cattle displayed an earlier and more extensive PrP^Sc^ accumulation in the trigeminal ganglion and in the optic nerve than L-BSE challenged cattle, whereas L-BSE infected cattle displayed a stronger involvement of the vagus nerve, the peripheral motoric nervous system and the musculoskeletal system. By transgenic mouse bioassay, we were also able to detect abundant amounts of BSE infectivity in the peripheral nerves of L-BSE infected cattle, but not to the same extent in the same samples collected from animals challenged with H-BSE. This may be partly due to the distinctly prolonged incubation times in transgenic mice after challenge with H-BSE as compared to C-BSE or L-BSE, possibly extending the incubation time in some experiments beyond the lifespan of the mice. This is in line with our results from parallel titration experiments of L-BSE and H-BSE in bovine PrP transgenic mice, where we found at least a tenfold increase in the LD_50_ titre for the L-BSE material as compared to H-BSE.

While H-BSE challenged cattle displayed a predominantly glial immunolabelling pattern and only to a lesser extent intraneuronal PrP^Sc^ accumulations, L-BSE affected cattle displayed a PrP^Sc^ distribution with a predominant intraneuronal immunolabelling pattern in the vicinity of the nucleus, confirming reports from earlier studies [29,35,40,41]. Histopathological analysis revealed that although all experimental animals were in a comparable clinical stage of the disease, L-BSE challenged cattle exhibited generally more and larger, sometimes locally confluent spongiform degenerations in contrast to H-type BSE affected cattle. The most striking differences were observed in the trigeminal tract nuclei in cattle at the end stage of the disease, and to a lesser extent also in the solitary tract nuclei and the DMNV.

These divergent PrP^Sc^ accumulation patterns and histopathological changes indicate different cell tropisms correlated with these BSE forms, as it has already been described for different scrapie strains in sheep [42]. We, therefore, postulate a different origin for both atypical BSE forms, in regard to the cell tropism and also in regards to the tissues of initial PrP^Sc^ propagation. We suggest that both atypical L- and H-BSE forms have their origin in the nervous system, while classical BSE ascends after oral (natural) infection from the gut via peripheral nerves to the brain [13,15]. L-BSE affected cattle BSE showed very high PrP^Sc^ concentrations in the neurons of all analysed brain regions with peaks in the rhinencephalon, midbrain and thalamus [39], in combination with the presence of large, partly confluent vacuoles, and also early PrP^Sc^ depositions in the vagus nerve and in the peripheral motoric nervous system. A primary PrP^Sc^ formation in the neurons of the CNS and possibly simultaneously in the PNS with a neuronal spread throughout the body seems a feasible scenario for this BSE form.

Meanwhile, H-BSE affected cattle displayed similarly even, albeit overall lower PrP^Sc^ signals in all brain regions [39], with a predominantly glial PrP^Sc^ deposition pattern. In this BSE form, the spinal cord, optic nerve, DRG and trigeminal ganglion were more prominently involved at early stages of the clinical disease. From this, we postulate that while L-BSE may first replicate solely in the neurons of infected cattle, H-BSE displays a strong tropism to glial cells within the CNS. Meanwhile, we observed a trend towards a stronger involvement of efferent fibres as compared to afferent fibres of the white matter of the spinal cord in H-BSE affected cattle, suggesting the CNS as the primary location of PrP^Sc^ propagation in the case of H-BSE.

Due to the at least 10-fold increased susceptibility of Tgbov XV mice to challenge with L-BSE as compared to H-BSE, the results of the mouse bioassay were not used for the direct comparison of the infectivity load.

## Conclusions

The PrP^Sc^ and infectivity distribution patterns of atypical BSE seem to be generally similar to that known for classical BSE, with a clear restriction to the central and peripheral nervous and the musculoskeletal system. Taken our results together, we postulate distinct cell tropisms and propagation pathways for both atypical BSE forms, which both have their putative location of origin in the central nervous system. While L-BSE strongly affects the neurons of the CNS followed by the PNS, H-BSE initially affects CNS and PNS glia and only at later stages spreads to neurons. This is in sharp contrast to what is well acknowledged for C-BSE, which is the uptake of the infectious agent in the digestive tract, followed by a neuronal transport to the CNS.

## Materials and methods

### Experimental animals

The details of the intracranial challenge study with atypical BSE have been published previously [25]. Eleven female Holstein-Frisian calves (6 month of age) had been inoculated intracranially with 1 ml of a 10% (w/v) brain homogenate derived from the two German atypical BSE cases identified at that time [32]. Six calves had been challenged with L-BSE and five with H-BSE. The animals were sacrificed after 5–16 months post infection (mpi). Only the one L-BSE challenged animal sacrificed at 5 mpi was still in the preclinical stage, while the other cattle were presenting mild to severe BSE-associated clinical symptoms. During necropsy, an extensive tissue sampling had been carried out under TSE sterile conditions, using a fresh single set of instruments for every tissue sample collected and following a specific sampling order.

One half of the samples was formalin-fixed for histological examinations while the other half was frozen (−20°C and −70°C) for biochemical analysis.

In this study, we describe the detailed analysis of 38 selected organ samples per animal (Tables 1 and 3). This selection included tissues from the central (CNS) and peripheral nervous system (PNS), the lymphoreticular system (LRS), the alimentary, respiratory and reproductive system as well as the musculoskeletal system.

The correlating tissue samples collected during an oral C-BSE pathogenesis study [13] had either been collected under identical conditions and analysed using the same methods, or were available for comparative analysis performed during this study.

### Biochemical analysis

First, the peripheral tissue samples were analysed for the presence of PrP^Sc^ using the IDEXX HerdChek® BSE enzyme immunoassay (EIA), which is one of the approved BSE rapid test systems. This test system is based on a PrP^Sc^-specific ligand immobilized on a microplate to bind the BSE antigen and it was performed according to the manufacturer’s instructions (short protocol).

For Western blot analysis of 10% tissue homogenates, we performed a PrP^Sc^ precipitation protocol using phosphotungstic acid with subsequent Western blot (PTA WB) and mAb L42 (r-biopharm, Art.-No. R8005) as a detection antibody, as previously described [17]. In order to enable quantification of positive Western blot signals, sample homogenates with positive results were re-analysed in serial dilutions from 1:2 down to 1:1024, and signal intensities were assessed using the VersaDoc camera with the Quantity One software (Bio-Rad, Art.-No.1709602).

To increase the sensitivity for the analysis of neuronal samples in order to decipher the origin and dissemination of the BSE prions in the three different BSE forms, selected samples were also analysed using an optimized protocol for the purification of PrP^Sc^ including an ultracentrifugation protocol followed by Western blot [10]. These samples representing the central and peripheral motoric nervous system as well as the sympathetic and parasympathetic autonomous nervous system (Supplementary Table 1) were collected from two animals each that had been sacrificed at the clinical end stage of H-BSE (RA 15, RA 16), L-BSE (RA 02, RA 04) and C-BSE (IT 22, IT 38) and one mock-infected cattle (KT 30).

### Histopathological and immunohistochemical analysis

Spongiform alterations were assessed by histopathological analysis of Haematoxylin-Eosin (HE) stained tissue sections. Tissue samples were also processed for immunohistochemical analysis as described previously [43], with some modifications. We used a serial section technique with two to five levels per block and a plane distance of about 24 µm between each level, in order to analyse a tissue depth of 150 to 200 µm [17,33]. Two PrP-specific monoclonal antibodies (mab) were used in a mab cocktail for immunohistochemical staining, which was mab 6C2 (Central Veterinary Institute Wageningen, Art.-No 6C2/200) and mab F99/97.6.1 (VMRD Inc., Art.-No F99/97.6.1). The pre-treatment for this mab cocktail included an incubation for 30 min in 98%-100% formic acid, inhibition of the endogenous peroxidase with 3% H_2_O_2_ (Merck) in methanol for 30 min, followed by an autoclave step for 20 min at 121°C in citrate buffer (pH 6,0). These primary antibodies were applied at a concentration of 0.72 µg/ml for mab 6C2 and 0.25 µg/ml for mab F99/97.6.1 in TBS (Tris buffered saline) with 10% goat serum and 0.03% sodium azide, and incubated for 2 h at room temperature. Negative control sections were treated with a solution of 10% goat serum in TBS with 0.03% sodium azide. As a secondary antibody system, we used the EnVision^TM^ reagent (Dako, Art.-No K4001) containing a peroxidase-conjugated polymer backbone. Incubation time on these sections was 30 min at room temperature. The slides were finally developed with diaminobenzidine tetrahydrochloride (DAB Peroxidase Substrate Kit, Vector Laboratories, Art.-No SK-4100) for 10 min and counterstained with Mayer`s haematoxylin for 15 min. All sections were examined by light microscopy.

In order to verify the results obtained using the mab cocktail, the samples collected from the central and peripheral motoric nervous system, the sympathetic and parasympathetic autonomous nervous system, as well as the musculoskeletal system were also analysed using mab 6C2 alone with the same protocol as described above.

### Mouse bioassay

In order to determine the sensitivity of Tgbov XV bovine PrP transgenic mice to both atypical BSE forms, we performed a parallel titration experiment, using serial tenfold dilutions from 10^−1^ to 10^−9^ of brain tissue from the initial H- and L-type BSE field cases diagnosed in Germany. Thirty microlitres per dilution were instilled intracranially into eight mice per group. The titres were calculated using the Logit Model with R, Version 2.15.2.

Selected samples that had been collected under TSE sterile conditions from the central and peripheral nervous system as well as from the musculoskeletal system of two animals challenged with L-BSE and sacrificed at 16 mpi (RA02, RA04) and two animals challenged with H-BSE and sacrificed at 16 mpi (RA15, RA16) were also analysed by a highly sensitive transgenic mouse bioassay [6]. Ten percent homogenates were prepared in 0.9% NaCl solution by aspiration through syringes and needles starting from 18G to 23G. These homogenates were then inoculated intracranially (30 µl i.c.) into 15 Tgbov XV mice per group. Mice were checked for their health status 3 times a week. Animals showing at least two clinical symptoms indicative of a BSE infection, such as hind limb paresis, abnormal tail tonus, behavioural changes and weight loss over several consecutive days, were sacrificed and brain samples were taken for diagnostic evaluation. Mouse brains were analysed in a PTA-WB as described above. Incubation times were calculated as the time between the inoculation and the death of the animal. All animals of an experiment that died more than 50 days post inoculation were tested for PrP^Sc^ accumulation, and only mice with positive results were included in the calculation of incubation times.
