# Supplementary figures and images for: Deciphering the BSE-type specific cell and tissue tropisms of atypical (H and L) and classical BSE
Source: Prion. 2019 Sep 3;13(1):160–72. doi: 10.1080/19336896.2019.1651180 (PMC6746549; doi:10.1080/19336896.2019.1651180)

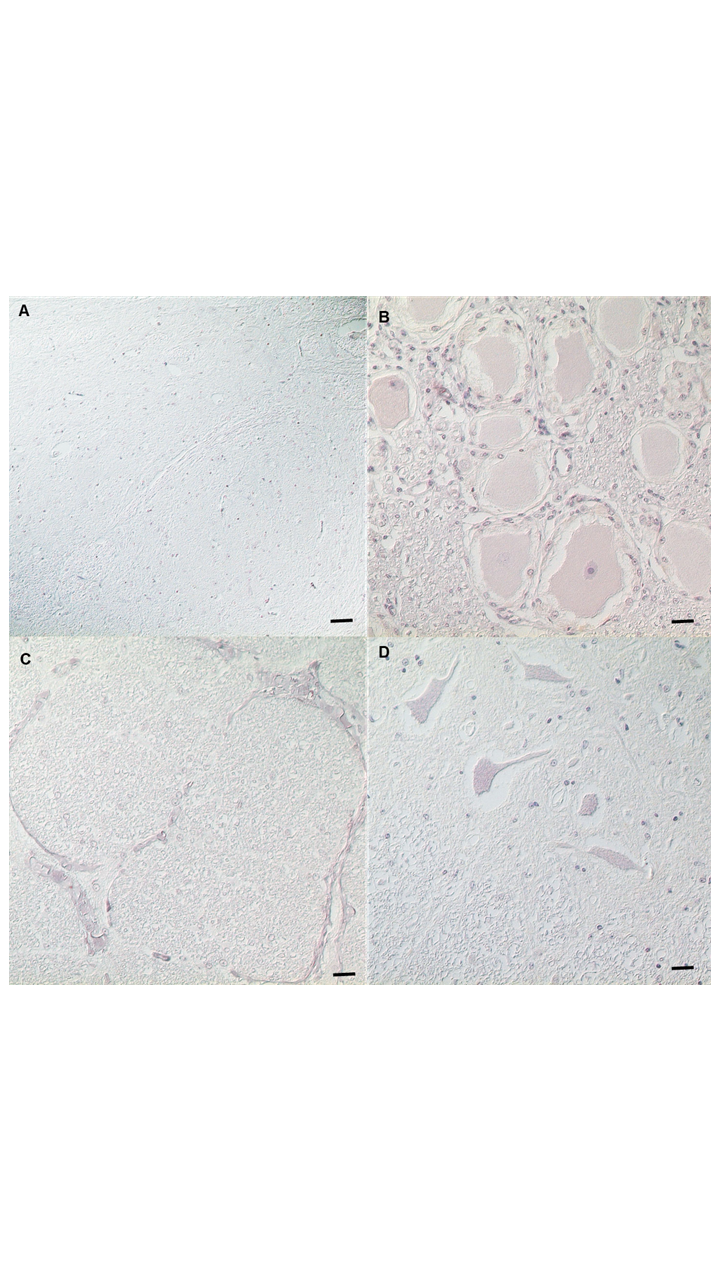

Supplement: Supplemental Material [file kprn-13-01-1651180-s001.tif]
