# Supplementary material for: Deciphering the BSE-type specific cell and tissue tropisms of atypical (H and L) and classical BSE
Source: Prion. 2019 Sep 3;13(1):160–72. doi: 10.1080/19336896.2019.1651180 (PMC6746549; doi:10.1080/19336896.2019.1651180)
Supplement: Supplemental Material [file kprn-13-01-1651180-s002.docx]

**Supplementary Table 1: Neuronal samples from the motoric and autonomous central and peripheral nervous system analysed by ultracentrifugation assay (UC-A), immunohistochemistry (IHC) and transgenic mouse bioassay (BA)**

| **Functional part of the nervous system** | **Tissue** | **L-BSE**  **Cattle ID RA02** | | | **L-BSE**  **Cattle ID RA04** | | | **H-BSE**  **Cattle ID RA15** | | | **H-BSE**  **Cattle ID RA16** | | |
| --- | --- | --- | --- | --- | --- | --- | --- | --- | --- | --- | --- | --- | --- |
|  |  | **UC-A** | **IHC** | **BA** | **UC-A** | **IHC** | **BA** | **UC-A** | **IHC** | **BA** | **UC-A** | **IHC** | **BA** |
| **Central Motoric Nervous System** | Spinal cord | + | + | n.d. | ++ | + | n.d. | + | (+) | n.d. | + | (+) | n.d. |
|  | Dorsal root ganglia | (+) | (+) | + | - | (+) | + | - | + | + | - | (+) | + |
|  | Trigeminal ganglion | - | - | + | (+) | - | + | + | (+) | + | + | (+) | + |
| **Peripheral Motoric Nervous System** | Radial nerve | - | - | n.d. | - | - | n.d. | - | - | n.d. | - | - | n.d. |
|  | Tibial nerve | - | - | n.d. | - | - | n.d. | - | - | n.d. | - | - | n.d. |
|  | Saphenous nerve | - | - | + | - | - | + | - | - | + | - | - | + |
| **Sympathetic Autonomous Nervous System** | Stellate ganglion | - | n.d. | + | - | - | + | - | - | + | - | - | + |
|  | Coeliac ganglion | - | - | n.d. | - | - | n.d. | - | n.d. | n.d. | - | n.d. | n.d. |
|  | Splanchnic nerves | - | - | n.d. | - | - | n.d. | - | - | n.d. | - | - | n.d. |
| **Parasympathetic Autonomous Nervous System** | Cervical vagus nerve | (+) | - | + | (+) | - | - | - | - | - | - | - | - |
|  | Nodose ganglion | + | - | n.d. | + | - | n.d. | + | - | n.d. | + | - | n.d. |

**Supplementary Table 2:** Parallel titration of H-, and L-BSE in Tg bovXV mice

| **Dilution** | **H-BSE**  **Attack rate /**  **mean incubation times (days)** | **L-BSE**  **Attack rate /**  **mean incubation times (days)** |
| --- | --- | --- |
| **10^-1^** | 7/8 (380 d) | 7 / 8 (175 d) |
| **10^-2^** | 7/8 (345 d) | 7 / 8 (208 d) |
| **10^-3^** | 6/8 (513 d) | 7 / 7 (243 d) |
| **10^-4^** | 3/8 (435 d) | 8 / 8 (288 d) |
| **10^-5^** | 0/7 (> 727 d) | 2 / 8 (341 d) |
| **10^-6^** | 1/8 (407 d) | 2 / 7 (642 d) |
| **10^-7^** | 2/8 (374 d) | 0 / 7 (> 677 d) |
| **10^-8^** | 0/8 (> 727 d) | 0 / 8 (> 605 d) |
| **10^-9^** | 0/8 (> 566 d) | 0 / 8 (> 727 d |
| **LD_50_ titre**  **(95% confidence interval)** | **10^-3.712^**  **(10^-4.517,-2.907^)** | **10^-4.767^**  **(10^-5.443,-4.090^)** |

LD_50_ titre in a C-BSE inoculum: 10−^5.730^ (10^−6.569^–10^−4.891^)
