# Supplementary material for: Deciphering the BSE-type specific cell and tissue tropisms of atypical (H and L) and classical BSE
Source: Prion. 2019 Sep 3;13(1):160–72. doi: 10.1080/19336896.2019.1651180 (PMC6746549; doi:10.1080/19336896.2019.1651180)
Supplement: Supplemental Material [file kprn-13-01-1651180-s003.docx]

**Supplementary Figure 1**

Immunohistochemistry of tissues of a mock-infected cattle showing no PrP^Sc^ immunoreactivity. A) obex, nucleus of the spinal tract of the trigeminal nerve. B) Trigeminal ganglion. C) N. opticus. D) spinal cord, bottom left white matter, upper right grey matter. Immunohistochemistry; monoclonal anti-PrP antibody cocktail (clones: 6C2 and F99/97.6.1); horseradish peroxidase-and polymer coupled secondary rabbit anti-mouse antibody (EnVision^TM^, DAKO); diaminobenzidine tetrahydrochloride (brown); Mayer`s hematoxylin counterstain (blue); Nomarski contrast; Bars A = 50 µm; B - D = 20 µm.
